# Response rates and minimal residual disease outcomes as potential surrogates for progression-free survival in newly diagnosed multiple myeloma

**DOI:** 10.1371/journal.pone.0267979

**Published:** 2022-05-12

**Authors:** Patrick Daniele, Carla Mamolo, Joseph C. Cappelleri, Timothy Bell, Alexander Neuhof, Gabriel Tremblay, Mihaela Musat, Anna Forsythe

**Affiliations:** 1 Purple Squirrel Economics, a Cytel Company, Montreal, Canada; 2 Pfizer Inc., Groton, Connecticut, United States of America; 3 Pfizer Inc., New York, New York, United States of America; 4 Pfizer Pharma GmbH, Berlin, Germany; 5 Purple Squirrel Economics, a Cytel Company, New York, New York, United States of America; Mayo Clinic, UNITED STATES

## Abstract

Progression-free survival (PFS) is a common primary endpoint in newly diagnosed multiple myeloma (NDMM). Patients with NDMM typically have longer PFS and are more likely to achieve minimal residual disease (MRD) or complete response (CR) compared to patients with relapsed or refractory multiple myeloma. Response-based surrogate endpoints may hold value given the longer follow-up time required to evaluate PFS in NDMM. In this work, systematic literature reviews of Medline, Embase, and Cochrane databases (2010-06/2020) and relevant congresses (2018–2020) were performed to identify randomized clinical trials (RCTs) and real-world studies in NDMM reporting median PFS and objective response. Associations between PFS and each response endpoint were evaluated using Pearson’s product-moment correlation weighted by sample size in each RCT arm. Unadjusted and adjusted weighted linear regression models were applied to estimate the gain in median PFS associated with each response endpoint. Statistically significant correlations were identified for median PFS with overall response rate (ORR; Pearson r = 0.59), CR (r = 0.48), stringent CR (sCR; r = 0.68), and MRD (r = 0.69). The unadjusted models estimated 0.50 (95% CI: 0.36, 0.64; p<0.001), 0.42 (95% CI: 0.25, 0.58; p<0.001), 1.05 (95% CI: 0.58, 1.52; p<0.001), and 0.35 (95% CI: 0.12, 0.58; p = 0.006) months of median PFS gained per point of ORR, CR, sCR, and MRD, respectively. Associations for median PFS remained statistically significant in models adjusted for age and treatment type with ORR (0.35, 95% CI: 0.21, 0.49; p<0.001), and adjusted for age and International Staging System risk stage with CR (0.29, 95% CI: 0.16, 0.41; p<0.001). Due to small sample size, adjusted models could not be constructed for sCR or MRD. Nevertheless, evidence of significant survival benefit (p<0.05) associated with MRD negativity and sCR was identified across real-world studies. These findings provide support for the use of response outcomes as surrogate endpoints to estimate PFS benefit in NDMM.

## Introduction

Multiple myeloma (MM) is the second most common hematologic malignancy, with approximately 32,270 new cases diagnosed each year in the US [1, 2]. In recent years, a range of options have been approved to treat patients with newly diagnosed multiple myeloma (NDMM) [3]. Proteasome inhibitors, immunomodulators, and monoclonal antibodies offer substantial improvements in remission rates and survival, particularly when used early in the disease course; however, the five-year survival rate of 55.6% between 2011 and 2017 suggests a substantial opportunity remains to improve patient outcomes [1, 2, 4–6]. While these gains are promising, they present challenges with respect to demonstrating the benefit of new therapeutics in a clinically relevant timeframe, since planned trials in the newly diagnosed patient population using survival endpoints now require much longer duration for mature data. Patients would benefit from timely access to efficacious treatments, if efficacy can be established earlier in the course of treatment, before lengthy follow-up.

Based on regulatory assessments, there is precedent for acceptance of response endpoints for accelerated approval pathways, but regulatory agencies expect additional confirmatory data from survival endpoints [7]. Likewise, payers and health technology assessment (HTA) agencies expect long-term outcome evidence for their assessments [8]. While overall survival (OS) is the gold standard in oncology trials, few studies in NDMM reach median OS at the time of primary endpoint publication or drug approval [4]. This is largely due to the efficacy of modern first-line treatments as well as the availability of numerous subsequent treatment options for multiple lines of life-prolonging therapy. In addition to the extended time for survival data maturation, additional lines of treatment and supportive care may confound the OS data from the initial trial [9, 10]. In the NDMM setting, median OS is over 9 years in several recent trials, including the CALGB 100104 study of lenalidomide maintenance therapy following autologous stem cell transplantation, which not only set a new benchmark but necessitated a meta-analysis of multiple phase 3 trials that employed progression-free survival (PFS) or time to progression (TTP) as the primary endpoint for regulatory approval [11, 12].

Because of design and timing challenges as well as the potential for confounding, recent studies have employed either PFS or, to a lesser extent, TTP in trials in the NDMM setting [4]. A meta-analysis intended to explore the relationship between PFS and OS among 21 MM randomized clinical trials (RCTs) demonstrated a moderate-to-strong correlation; however, recent exceptions have been noted [13, 14]. Regardless, PFS is the most common primary endpoint in contemporary clinical trials in the NDMM setting. Nonetheless, the sample size and follow-up requirements to demonstrate significant improvements present challenges that have increased interest in identifying viable surrogate endpoints, such as overall response rate (ORR), complete response (CR), stringent complete response (sCR), or minimal residual disease (MRD), all of which are clinically relevant but have not been completely explored in patients with NDMM [4]. Patients with NDMM are more likely to achieve negative MRD status or CR than patients with relapsed or refractory disease, in addition to longer PFS [15]. In an analysis of recent RCTs reporting both MRD and PFS in the NDMM setting, median PFS of included studies ranged from 18.1 months to 56.3 months; however, median PFS in newly diagnosed, MRD-negative patient populations as long as 110 months have been reported [16–18]. Coupled with the fact that MRD negativity is recognized as a strong prognostic factor for both OS and PFS, its inclusion in trials and use in the clinical setting to monitor patients and help inform treatment decisions is on the rise [15]. Response-related endpoints and MRD status allow for a more rapid assessment of drug activity in the population of interest and may serve as early efficacy indicators despite not measuring survival directly. Recent meta-analyses have supported the utility of MRD negativity as a surrogate for PFS and OS in patients with NDMM [18, 19].

We therefore investigated the role of response endpoints as surrogates for survival endpoints in NDMM RCTs, while also assessing the literature-reported association of MRD with survival in real-world studies.

## Methods

### Literature search and article selection

A systematic literature review (SLR) of Medical Literature Analysis and Retrieval System Online (MEDLINE®), Excerpta Medica Database (Embase®) and Cochrane collaboration was performed according to Preferred Reporting Items for Systematic Reviews and Meta-Analyses guidelines to retrieve articles published between 2010 and the search date of 26 July 2020 [20]. Additionally, abstracts from relevant congresses from 2018 to 2020 as well as bibliographies of systematic reviews and meta-analyses were reviewed to ensure all relevant studies were captured. The SLR scope was defined in terms of the patient population, intervention, comparators, outcome measures, and study design (PICOS statement; S1 Table). The SLR search strategy is provided in S2 Table. Eligible records were English-language interventional studies reporting ORR, CR, sCR, and MRD, in addition to median PFS associated with induction therapy with regimens including only approved treatments (bortezomib, lenalidomide, carfilzomib, ixazomib, daratumumab, thalidomide, and/or melphalan) in the NDMM setting. RCTs were included regardless of blinding and single-arm studies were also included. Evidence from observational studies (both prospective and retrospective), case series, and case reports was excluded. From included studies, all reported endpoints, study designs, and patient characteristics were extracted and used for the statistical analysis. In addition, a separate SLR for real-world evidence (RWE) studies was conducted using similar search parameters and constraints to further investigate the association between MRD and survival.

### Statistical analysis

Results of the SLR were used to construct analytic datasets based on individual study arms. A meta-analysis was then performed on the aggregate data to estimate correlations and construct regression models weighted by study sample sizes [21]. All reported endpoints were considered as possible surrogates for median PFS since median OS generally was not reached in NDMM studies. Correlations between median PFS and each surrogate endpoint were estimated using Pearson’s product–moment correlation weighted by sample size in each study arm. The strength of the association was then categorized based the recommendations of the Institute of Quality and Efficiency in Health Care: low correlation (r ≤ 0.7), medium strength correlation (0.7 < r < 0.85) and high correlation (r ≥ 0.85) [22]. Survival gains associated with improvement in each surrogate endpoint were estimated using the following weighted linear regression models:

Unadjusted: Median PFS Months = β_0_ + β_1_Surrogate + e
Adjusted: Median PFS Months = β_0_ + β_1_Surrogate + β_i_X_i_+ e

where β_0_ is the intercept, β_1_ is the coefficient per percentage point of each surrogate value (% ORR, and % CR), X_i_ and β_i_ are observed values and coefficients for covariates, respectively.

Diagnostic plots were used to verify the assumptions of the linear regression models. Moreover, multivariable models were constructed to assess covariates for adjustment, including key prognostic patient characteristics such as median age, sex, eligibility for transplant (ineligible only vs eligible and ineligible), International Staging System (ISS) risk stage 3 percent, and high cytogenetic risk. Trial design (RCT vs single arm), treatment phase (induction + maintenance vs induction only), and treatment type were also included. Due to the similarity of outcomes between lenalidomide (LEN) and bortezomib (BOR)-based therapies in contrast to other interventions, treatment type was dichotomized to LEN/BOR based vs other. These factors were considered based on evidence of their prognostic value [23, 24] and data availability. Optimal models were selected based on model fit statistics such as Akaike information criteria (AIC), Bayesian information criteria (BIC), R^2^ and adjusted R^2^, and mean squared error. All statistical analyses were performed using RStudio version 1.2.5033.

## Results

### Search results

**Fig 1:**
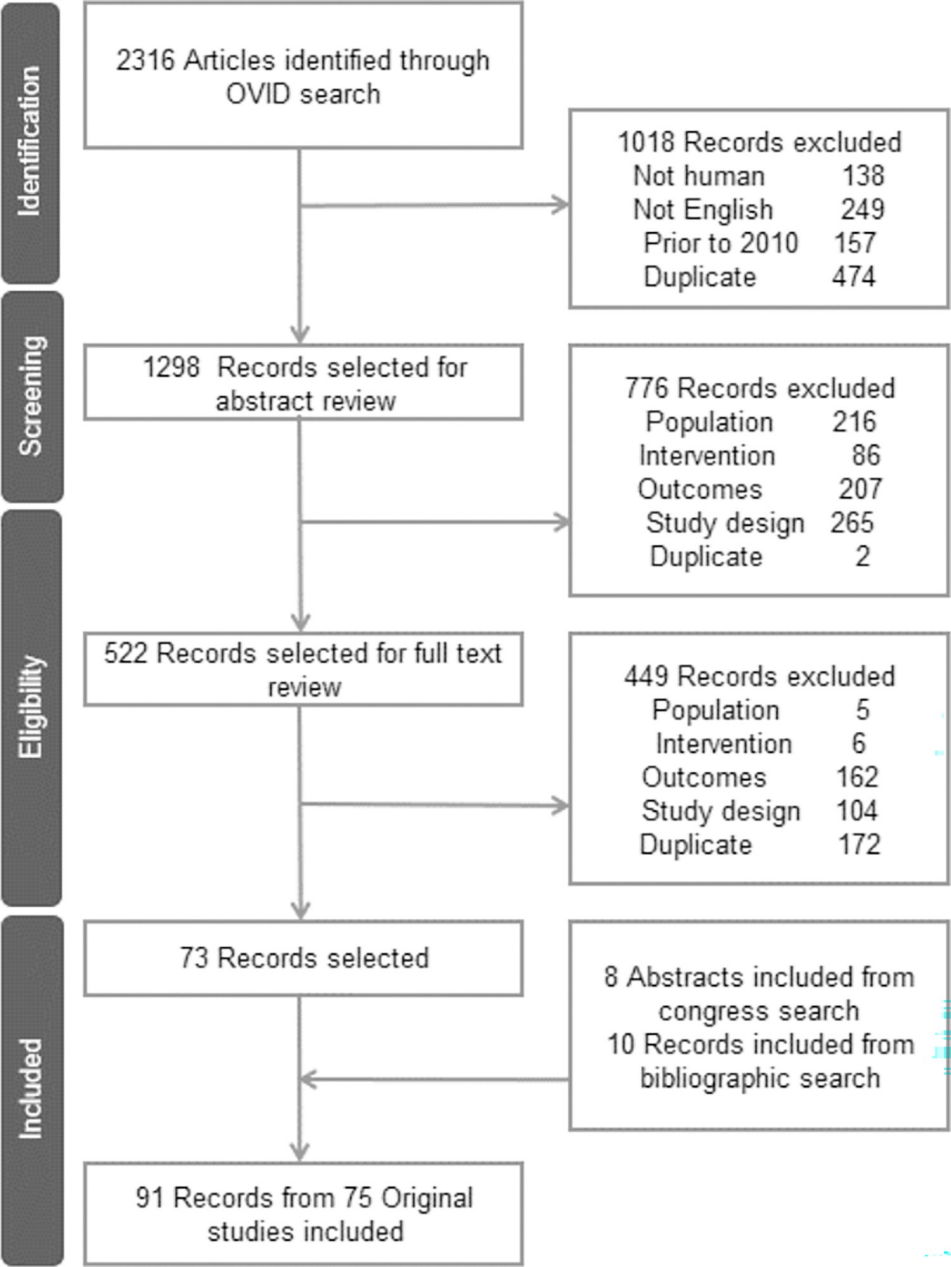
Article identification and selection: Preferred reporting items for systematic reviews and meta-analyses from the systematic literature review of clinical trials in newly diagnosed multiple myeloma.

The SLR identified 91 individual abstracts and manuscripts from 75 original studies consisting of 19 RCTs, 15 of which were phase 3 studies, and 7 single arm studies (Fig 1). Data were extracted from 137 study arms (N = 23,352) with populations ranging from 11 to 1,021 patients per arm. Median PFS was reported in 132 study arms, and reporting of the other endpoints of interest varied, with 124 reporting ORR, 122 CR, 104 very good partial response (VGPR), 31 sCR, and 31 MRD. The other endpoints expressed in terms of months, duration of response (DOR), time to next therapy (TNT), event-free survival (EFS), and time to progression (TTP), were reported in 25 or fewer study arms (Table 1). Based on the available endpoint reporting in the selected studies, ORR, CR, and VGPR had sufficient data for surrogate endpoint validation. VGPR alone does not include patients who reach CR; therefore, ORR is a preferable response-based surrogate endpoint unless a multi-level association could be constructed which factors in the level of response; as such, results for VGPR are not reported. Because of the clinical interest in sCR and MRD, exploratory analyses were performed for these endpoints.

**Table 1:** Characteristics and endpoint availability in identified studies.

**Study Characteristics**	**N = 101**	**NA**
**Study year**, n (%)		0
• Pre-2015	41 (40.6)	
• Post-2015	60 (59.4)	
**Age** (years), median (range)	71.0 (52.0, 79.0)	18
**Male** (%),	53.5 (6.3)	21
**ECOG PS 0** (%), mean (SD)	35.6 (9.5)	75
**ISS Stage 3** (%), mean (SD)	31.6 (11.8)	20
**High cytogenetic risk** (%), mean (SD)	23.3 (18.8)	52
**Transplant ineligible only**, n (%)	49 (48.5)	13
**Treatment phase**		0
• Induction only	22 (21.8)	
• Maintenance	49 (48.5)	
• Other	30 (29.7)	
**Treatment type**		0
• LEN/BOR	61 (60.4)	
• Other	40 (39.6)	
**Endpoint Availability**	**Available**	**NA**
ORR	89	12
CR	87	14
VGPR	73	28
sCR	20	81
MRD	14	87
DOR	20	89
TNT	15	86
TTP	12	89

CI, confidence interval; CR, complete response; BOR, bortezomib; DOR, duration of response; ECOG PS, Eastern Cooperative Oncology Group performance status; ISS, International Staging System; LEN, lenalidomide; MRD, minimal residual disease; NA; not available/missing; ORR, overall response rate; PFS, progression-free survival; sCR, stringent complete response; SD, standard deviation; TNT, time to next therapy; TTP, time to progression; VGPR, very good partial response.

### Statistical analysis

**Fig 2:**
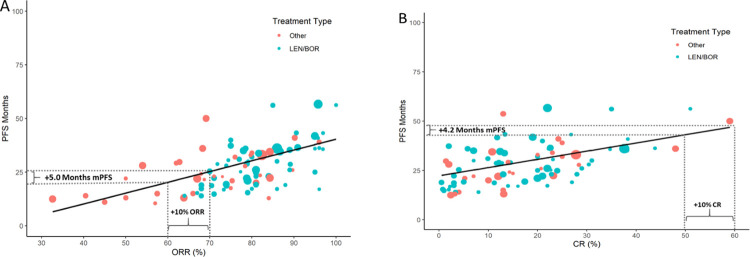
Scatterplots of CR and ORR on median PFS months. Scatterplots depict the unadjusted linear model overlayed on the raw data, with point size indicating the relative sample size of each study arm for A) PFS vs ORR and B) PFS vs CR. Dashed lines demonstrate the interpretation of the unadjusted model coefficients. CI, confidence interval; CR, complete response; BOR, bortezomib; LEN, lenalidomide; ORR, overall response rate; PFS, progression-free survival.

The correlation between ORR and median PFS was low (weighted Pearson r = 0.59). The unadjusted linear model demonstrated reasonable fit to the data weighted by sample size (Fig 2A). LEN/BOR-based therapies were generally associated with higher ORR and longer survival than other therapies. According to the unadjusted model, the association was significant (p<0.001), with each percentage point increase in ORR predicting an average median PFS gain of 0.50 months (95% confidence interval CI: 0.36, 0.64). When the model was adjusted for age and treatment type (LEN/BOR vs other), the gain was reduced to 0.35 months (95% CI: 0.21, 0.49).

Rates of CR were generally low and did not differ substantially between LEN/BOR-based regimens and other therapies. The unadjusted linear model demonstrated reasonable fit to the data weighted by sample size and the correlation between CR and median PFS was low (weighted Pearson r = 0.48) (Fig 2B). Each percentage point increase in CR predicted a median PFS gain of 0.42 months (95% CI: 0.25, 0.58) on average according to the unadjusted model (Table 2). After adjustment for age and ISS risk stage, the associations remained significant but were attenuated to 0.29 months (95% CI: 0.16, 0.41) of median PFS for each point of CR.

**Table 2:** Modeling results for ORR and CR as predictors of median PFS.

Category	Increase in median PFS months per % increase in ORR (95% CI)	p-value	R^2^	Increase in median PFS months per % increase in CR (95% CI)	p-value	R^2^
**Unadjusted**	0.50 (0.36, 0.64)	<0.001	0.37	0.42 (0.25, 0.58)	<0.001	0.22
**Adjusted** ^**a**^	0.35 (0.21, 0.49)	<0.001	0.63	0.29 (0.16, 0.41)	<0.001	0.632

^a^Adjusted age and treatment type (LEN/BOR vs other) for ORR model and age and ISS risk stage for CR model.

CI, confidence interval; CR, complete response; BOR, bortezomib; ISS, International Staging System; LEN, lenalidomide; ORR, overall response rate; PFS, progression-free survival

### Exploratory analyses

**Fig 3:**
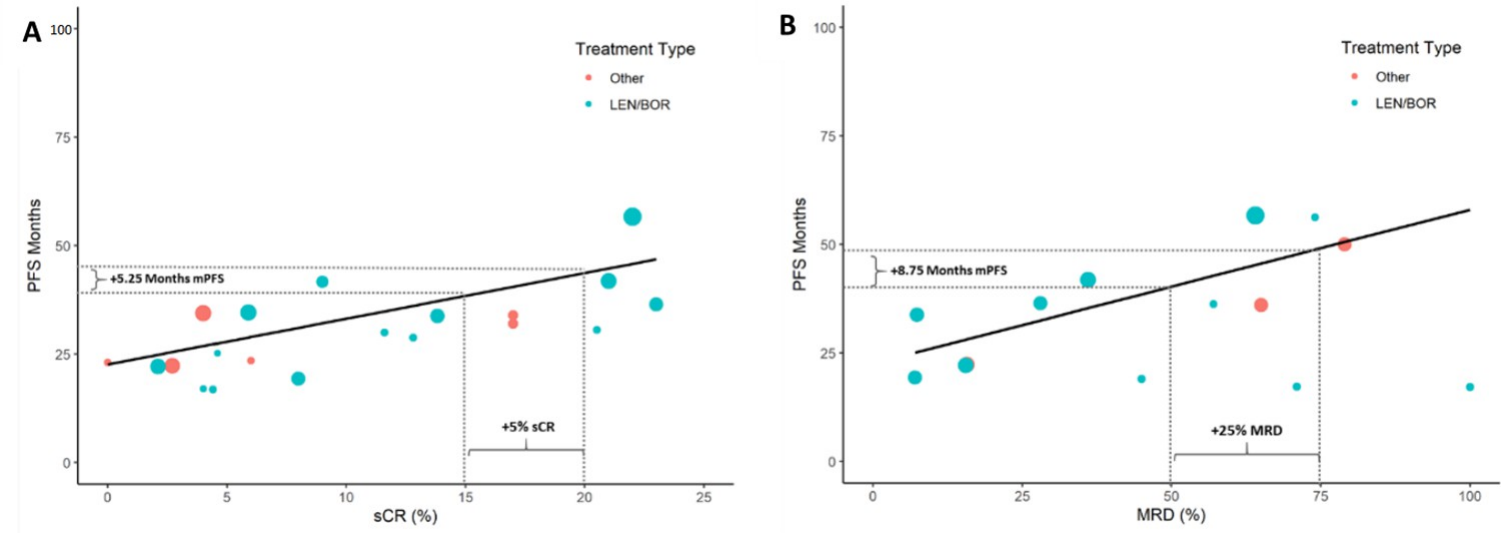
Scatterplots of sCR and MRD on median PFS months. Scatterplots depict the unadjusted linear model overlayed on the raw data, with point size indicating the relative sample size of each study arm for A) PFS vs sCR (%) and B) PFS vs MRD (%). Dashed lines demonstrate the interpretation of the unadjusted model coefficients. CI, confidence interval; BOR, bortezomib; LEN, lenalidomide; MRD, minimal residual disease; ORR, overall response rate; PFS, progression-free survival; sCR, stringent complete response.

Despite limited data, analyses of sCR and MRD were conducted due to current interest in—and clinical relevance of—these endpoints (Fig 3, Table 3). Correlations with median PFS were low for both sCR (weighted Pearson r = 0.68) and MRD (weighted Pearson r = 0.69); however, the associations were statistically significant. Increased sCR and MRD both predict significantly longer median PFS, with unadjusted models predicting an additional 5.25 months and 8.75 months of median PFS per 5% increase in sCR and 25% increase in MRD, respectively. Due to limited sample size, adjusted models could not be constructed.

**Table 3:** Modeling results for sCR and MRD as predictors of median PFS.

Response endpoint	Increase in median PFS per % increase in response endpoint, months (95% CI)	p-value	R^2^
**sCR (%)**	1.05 (0.58, 1.52)	<0.001	0.53
**MRD (%)**	0.35 (0.12, 0.58)	0.006	0.44

CI, confidence interval; BOR, bortezomib; LEN, lenalidomide; MRD, minimal residual disease; PFS, progression-free survival; sCR, stringent complete response

The RWE SLR identified 49 relevant studies reporting on NDMM, representing a total of 10,082 patients. The majority were retrospective (N = 37), and most were single center (N = 30). Substantial heterogeneity with respect to rates of sCR (range: 0 to 46%; N = 25 studies) and MRD (range:10% to 75%; N = 19 studies) was observed in identified studies. Among 20 studies that investigated correlations of OS/PFS with MRD/sCR, 12 reported a significant survival benefit (p<0.05) associated with MRD negativity, and two demonstrated significantly increased survival among patients with sCR.

## Discussion

This meta-analysis revealed statistically significant associations, with low correlation, between ORR and CR and median PFS in NDMM, providing evidence to support the use of these outcomes as surrogate endpoints to demonstrate PFS benefit in this population. According to our adjusted model analysis, a 10% ORR or CR increase predicts an incremental median PFS gain of 3.5 months and 2.9 months, respectively. While OS is the gold standard of primary endpoints for demonstrating a survival benefit in oncology trials, PFS is a well-accepted surrogate for OS that can provide more meaningful information on the impact of a first-line treatment [4, 7, 13, 25]. Because patients often survive for several years following initial treatment, median OS is often not reached in the NDMM setting and is subject to the confounding effect of numerous subsequent therapies because patients generally receive many rounds of therapy. Among identified studies in our SLR, a smaller total number reported OS data than PFS data because the median OS was not reached at the time of study publication. Based on the limited results (39 study arms) reporting median OS, our exploratory analysis of reported endpoints and OS showed a low correlation between median OS and CR. This finding may be attributable to reporting bias, wherein studies with high CR were not identified as median OS was not reached.

Recently, both sCR and MRD have emerged as clinically relevant endpoints of increasing interest in MM [18, 19, 26]. Our exploratory analysis of sCR and MRD demonstrated low correlations with statistically significant associations between both endpoints and median PFS, but adjusted models could not be constructed for sCR or MRD due to small sample size. For MRD in particular, the thresholds defined and reported in the identified studies varied from 10^−4^ (n = 7 studies) to 10^−6^ (n = 5 studies) and also were derived from differing measurement techniques. These factors, coupled with limited reporting, prevented us from performing additional sensitivity analyses or constructing a multivariable model that adjusted for the level of MRD negativity or measurement technique. This is an area for additional research as such data become available. An examination of RWE supports both the increasing recognition of these outcomes as treatment goals and their association with survival benefit; moreover, the search findings were consistent with the results of our analysis, providing further support for the utility of these endpoints as viable surrogates for PFS in NDMM.

The results of RCT findings and this analysis are aligned with the evidence in other types of cancer. Between 2006 and 2018, the U.S. Food and Drug Administration approved 59 therapies for 85 adult oncology indications on the basis of response rates [27]. Response rates have been associated with improved survival and have demonstrated promise as surrogate markers for survival in other cancers, including acute myeloid leukemia, renal cell carcinoma, and non-small cell lung, breast, and colorectal cancer [28–32]. According to an SLR of meta-analyses assessing response endpoints as surrogates for PFS or OS, there was wide variation [33], underscoring the need for additional studies investigating the relationship between relevant endpoints in specific indications.

While this study presents a comprehensive assessment of response endpoints as surrogates for survival outcomes, it has several limitations. Surrogate endpoint validation in each population was limited by small sample sizes and incomplete reporting of endpoints and patient characteristics. Several important prognostic variables such as Eastern Cooperative Oncology Group performance status and cytogenetic risk were not reported consistently, which limited variable selection for adjusted regression models. Furthermore, certain variables, such as high cytogenetic risk and MRD negativity were defined and measured differently across trials. With respect to high-risk cytogenetics, most studies included 17p-, t4;14, and t14;16; however, others had broader definitions including amp1q21, 13q-, 1p-, 1q+. This may have led to variability that we were unable to adjust for in our analyses due to limited sample size for the various definitions of MRD negativity and high-risk cytogenetics. The possibility of unmeasured confounding remains if factors associated with both surrogate endpoints and survival were unreported or imbalanced between trials and/or study arms. As a result, the conclusions of our study remain limited to the available studies with complete reporting of patient baseline characteristics. While treatment type was considered in the adjusted models, there was insufficient data with respect to chimeric antigen receptor T-cell (CAR T-cell) therapy to account for the possible disease-modifying effect of this potentially transformative treatment [34, 35]. Finally, analyses were conducted using aggregate data, and the observed correlations between intermediate endpoints and PFS may not hold at the individual patient level (ie, ecological fallacy) [36].

We identified low correlation but statistically significant associations between key response endpoints and PFS. According to IQWiG definitions, any R value below 0.7 constitutes a low correlation. The stringency of this threshold is such that the vast majority of identified surrogate endpoint studies do not meet this criteria, including surrogate endpoints that have been accepted by the U.S. Food and Drug Administration for drug licensure or approval [7, 33, 37].

In an SLR of endpoints used in MM RCTs by Mohyuddin et al (2021), only 10 out of 76 (13.2%) phase 3 studies in the NDMM setting employed OS as a primary endpoint; PFS was the most common primary endpoint [38]. Response rate was the most commonly used primary endpoint in identified phase 2 studies [38]. Because of the extended PFS observed in studies of patients with NDMM, a requirement for mature PFS data places a substantial burden on patients who are awaiting access to new therapies. Response endpoints have a long-standing role in allowing for accelerated assessment and approval of promising new therapies in oncology, with the expectation of confirmatory trials that will show evidence of benefit according to survival endpoints. The results of this comprehensive analysis demonstrated a significant association between surrogate endpoints, including ORR and CR, and PFS in NDMM. However, as response rates are already quite high in NDMM, there may be a decreasing margin for improvement remaining which motivates a shift toward more stringent measures of response such as MRD and sCR as surrogate endpoints.

## Supporting information

S1 Table SLR PICOS inclusion/exclusion criteria. (DOCX)

S2 Table Search strategy for the RCT SLR. (DOCX)
