# Supplementary material for: Response rates and minimal residual disease outcomes as potential surrogates for progression-free survival in newly diagnosed multiple myeloma
Source: PLoS One. 2022 May 12;17(5):e0267979. doi: 10.1371/journal.pone.0267979 (PMC9098007; doi:10.1371/journal.pone.0267979)
Supplement: S1 Table — (DOCX) [file pone.0267979.s001.docx]

**S1 Table. SLR PICOS inclusion/exclusion criteria.**

| Element | Inclusion criteria | Exclusion criteria |
| --- | --- | --- |
| Patient population | - Patients diagnosed with newly diagnosed MM | - Non-human - Patients with RRMM - Patients with asymptomatic MM/smoldering MM - Patients with other cancer types |
| Intervention and Comparators | - Induction treatment with: - Bortezomib - Lenalidomide - Carfilzomib - Ixazomib - Daratumumab - Thalidomide - Melphalan | - Radiotherapy - Surgery - Palliative care - 2L+ therapy - Stem cell transplantation - Studies reporting consolidation or maintenance treatment only (without induction) |
| Outcomes measures | - OS - PFS - Response rates (ORR/CR/sCR) - TTP - MRD | - Studies not including at least one of the outcomes listed in the Inclusion Criteria |
| Study design | - RCTs - Single-arm pivotal (phase 2, phase 3) trials - Non-randomized prospective interventional pivotal studies - Sub-group analyses of previously published studies - Systematic reviews, meta-analyses and indirect comparisons (for cross-checking only) - Pooled analyses (for cross-checking only) | - Non-human/pre-clinical studies - Reviews/ editorials/notes/comments/letters - Non-interventional studies - Retrospective studies - Observational studies - Phase 1 dose escalation study or pharmacokinetics study - Case reports/series |
| Restrictions | - English language - Year limitation: 2010 to current | - Non-English language studies - Case reports |

CR, complete response; L, line; MRD: minimal residual disease; ORR, overall response rate; OS, overall survival; PFS, progression-free survival; RCT, randomized clinical trial; sCR, stringent complete response; TTP, time to progression.
