# Supplementary material for: Response rates and minimal residual disease outcomes as potential surrogates for progression-free survival in newly diagnosed multiple myeloma
Source: PLoS One. 2022 May 12;17(5):e0267979. doi: 10.1371/journal.pone.0267979 (PMC9098007; doi:10.1371/journal.pone.0267979)
Supplement: S2 Table — (DOCX) [file pone.0267979.s002.docx]

**S2 Table. search strategy for the RCT SLR.**

| **Search conducted** | **26-Jul-2020** | | | |
| --- | --- | --- | --- | --- |
| **Databases searched** | EBM Reviews - Cochrane Database of Systematic Reviews 2005 to July 24, 2020, EBM Reviews - ACP Journal Club 1991 to June 2020, EBM Reviews - Database of Abstracts of Reviews of Effects 1st Quarter 2016, EBM Reviews - Cochrane Clinical Answers July 2020, EBM Reviews - Cochrane Central Register of Controlled Trials June 2020, EBM Reviews - Cochrane Methodology Register 3rd Quarter 2012, EBM Reviews - Health Technology Assessment 4th Quarter 2016, EBM Reviews - NHS Economic Evaluation Database 1st Quarter 2016, Econlit 1886 to July 16, 2020, Embase 1974 to 2020 July 23, Ovid MEDLINE(R) and Epub Ahead of Print, In-Process & Other Non-Indexed Citations, Daily and Versions(R) 1946 to July 23, 2020 | | | |
|  |  |  | **Term** | **Hits** |
| **Disease: MM** | Medline | 1 | multiple myeloma/ | 117,648 |
|  | Embase | 2 | myeloma/ or multiple myeloma/ | 127,599 |
|  | All | 3 | (myeloma$ or multiple-myeloma$ or Kahler$).ti,ab. | 140,098 |
| **Studies in MM** |  | **4** | **or/1-3** | **167,173** |
| **Newly diagnosed** | All | 5 | (((new$ or first) adj2 diagnos$) or NDMM).mp,af,tw. | 316,610 |
|  |  | 6 | (untreat$ or (no adj2 prior) or naive or treatment-naive or treatmentnaive).mp,af,tw. | 807,803 |
|  |  | 7 | (first line or first-line or firstline or 1st-line or 1st line or 1stline or 1L or 1-L or "1 L" or 1st-L or 1stL or 1st L or frontline or front-line or front line or initial treatment$ or initial therap$ or upfront treatment$ or upfront therap$).mp,af,tw. | 396,644 |
|  |  | 8 | or/5-7 | 1,459,868 |
| **Studies in NDMM** |  | **9** | **4 and 8** | **20,424** |
| **Interventions** | Medline | 10 | Bortezomib/ or Lenalidomide/ | 49,193 |
|  | Embase | 11 | bortezomib/ or lenalidomide/ or ixazomib/ or daratumumab/ or carfilzomib/ | 51,379 |
|  | All | 12 | (bortezomib or velcade$ or lenalidomide or revlimid$ or revimid$ or ixazomib or ninlaro$ or daratumumab or darzalex$ or carfilzomib or kyprolis$).ti,ab | 42,651 |
|  |  | 13 | or/10-12 | 60,478 |
| **Studies in treated NDMM** |  | **14** | **9 and 13** | **9,563** |
| **Interventional studies** | Medline | 15 | exp Randomized Controlled Trial/ or exp Random Allocation/ or exp randomization/ | 1,298,830 |
|  |  | 16 | exp Placebos/ | 411,452 |
| <https://www.nice.org.uk/guidance/ng50/documents/search-strategies> |  | 17 | exp Double-Blind Method/ or exp Single-Blind Method/ | 556,398 |
|  |  | 18 | exp clinical trial/ or exp clinical trial, phase ii/ or exp clinical trial, phase iii/ or exp controlled clinical trial/ | 2,371,055 |
|  |  | 19 | exp controlled clinical trials as topic/ or exp Randomized Controlled Trials as Topic/ or exp clinical trials as topic/ | 713,071 |
|  |  | 20 | exp Multicenter Study/ | 532,643 |
|  | Embase | 21 | exp Randomized Controlled Trial/ or exp Random Allocation/ or exp randomization/ | 1,298,830 |
|  |  | 22 | exp placebo/ | 352,469 |
|  |  | 23 | exp double blind procedure/ or exp single blind procedure/ or exp crossover procedure/ | 251,954 |
|  |  | 24 | exp clinical trial/ or exp phase 2 clinical trial/ or exp phase 3 clinical trial/ or exp controlled clinical trial/ | 2,371,055 |
|  |  | 25 | exp "controlled clinical trial (topic)"/ or exp "clinical trial (topic)"/ or exp "randomized controlled trial (topic)"/ | 327,319 |
|  |  | 26 | exp multicenter Study/ | 532,643 |
|  | All | 27 | randomized controlled trial.pt. | 1,008,175 |
|  |  | 28 | controlled clinical trial.pt. | 185,367 |
|  |  | 29 | random$.ti,ab,kw. | 3,789,715 |
|  |  | 30 | blind$.ti,ab,kw. | 1,071,964 |
|  |  | 31 | (placebo$ or assign$ or allocat$ or volunteer$).ti,ab,kw. | 2,446,733 |
|  |  | 32 | (parallel$ or factorial$ or crossover$ or cross over$).ti,ab,kw. | 1,096,089 |
|  |  | 33 | ('phase 3' or 'phase 2' or 'phase III' or 'phase II').af. | 531,747 |
|  |  | 34 | ((single or double or triple) adj3 (blind$ or mask$ or dummy)).af. | 941,209 |
|  |  | 35 | ('double-blind' or 'double-blinded').af. | 792,963 |
|  |  | 36 | (open label or open-label).af. | 192,468 |
|  |  | 37 | ("single arm" or "single-arm" or "single group" or "single-group").ti,ab. | 35,864 |
| **SLR** | Medline | 38 | exp Meta-Analysis/ or exp Meta-Analysis as Topic/ or exp "Systematic Review"/ | 596,172 |
| <https://www.nice.org.uk/guidance/ng50/documents/search-strategies> | Embase | 39 | exp meta analysis/ or exp "meta analysis (topic)"/ or exp "systematic review"/ | 579,360 |
|  | All | 40 | (meta analy$ or meta-analy$ or metanaly$ or metaanaly$).ti,ab. | 449,154 |
|  |  | 41 | ((systematic$ or evidence$) adj3 (review$ or overview$)).ti,ab. | 554,890 |
|  |  | 42 | ((multiple treatment$ or indirect or mixed) adj2 comparison$).ti,ab. | 9,290 |
|  |  | 43 | or/15-42 | 8,665,203 |
| **Interventional studies or SLRs in treated NDMM** | All | **44** | **14 and 43** | **5,623** |
| **Relapsed or refractory** |  | 45 | (relap$ OR refract$ OR reoccur$ OR reocur$ OR (re adj2 occur) OR (re adj2 ocur$) OR recurren$ OR salvage$ or RRMM).ti,ab. | 2,346,539 |
| **Irrelevant study design** |  | 46 | (addresses or bibliography or biography or case report or comment or congresses or consensus development conference or duplicate publication or editorial or guideline or interview or lectures or letter or monograph or news or practice guideline or "review literature" or "review of reported cases" or review, academic or review, multicase or review, tutorial or twin study).pt. | 4,108,239 |
|  |  | 47 | (animals/ not (humans/ and animals/)) or (animal/ not (human/ and animal/)) | 5,807,181 |
|  |  | 48 | case report/ or case reports/ | 4,610,195 |
|  |  | 49 | epidemiologic studies/ or epidemiology/ | 229,037 |
|  |  | 50 | exp case-control studies/ or exp case control study/ | 1,283,791 |
|  |  | 51 | exp cross-sectional studies/ or exp cross-sectional study/ | 694,893 |
|  |  | 52 | retrospective studies/ or retrospective study/ | 1,778,671 |
|  |  | 53 | observational study/ | 284,165 |
|  |  | 54 | (case control or case-control or retrospective or cross sectional or cross-sectional or observational).ti,ab. | 2,871,567 |
|  |  | 55 | (registry or claims or survey or chart review or real world or real-world or audit).ti,ab. | 1,932,903 |
|  |  | 56 | cost-benefit analysis/ or "cost effectiveness analysis"/ or "cost minimization analysis"/ or "cost benefit analysis"/ or "cost utility analysis"/ | 378,835 |
|  |  | 57 | (cost$ adj2 (effective$ or utilit$ or benefit$ or minimi$ or consequence$)).ti,ab | 439,537 |
|  |  | 58 | (CEA or CMA or CBA or CUA).ti,ab. | 95,263 |
|  |  | 59 | or/45-58 | 21,460,012 |
|  |  | 60 | 44 not 59 | 2,316 |
| **Limits** |  | 61 | limit 60 to english language | 2,067 |
|  |  | 62 | limit 61 to humans | 1,929 |
|  |  | 63 | limit 62 to yr="2010 -Current" | 1,772 |
| **FINAL interventional studies or SLR in treated NDMM** |  | **64** | **Deduplicate** | **1,298** |

CBA, cost-benefit analysis; CEA, cost-effectiveness analysis; CMA, cost-minimization analysis; CR, complete response; CUA, cost-utility analysis; L, line; NDMM, newly diagnosed multiple myeloma; RCT, randomized clinical trial; SLR, systematic literature review.
